# Annual acknowledgement of reviewers

**DOI:** 10.1186/1471-2121-15-3

**Published:** 2014-01-28

**Authors:** Tim Sands

**Affiliations:** 1 BioMed Central, Floor 6, 236 Gray's Inn Road, London WC1X 8HB, UK

## Abstract

**Contributing reviewers:**

The editors of BMC Cell Biology would like to thank all our reviewers who have contributed to the journal in Volume 14 (2013).

## 

Dieter Adam

Germany

Stephen Alway

USA

Nagabhushana Anathamurthy

India

Michael Anderson

USA

Hitoshi Ando

Japan

Hiroshi Asahara

Japan

Giuseppe Astori

Italy

Jose Bayascas

Spain

Allison Berrier

USA

Magdalena Bezanilla

USA

Alfonso Blanco

Ireland

Vilmante Borutaite

Lithuania

Marina Bouche

Italy

François-Yves Bouget

France

Nathan Bowen

USA

Claudio Brancolini

Italy

Nickolay Brustovetsky

USA

Carolyn Anne Carr

UK

James Castelli-Gair Hombría

Spain

Yang Chen

China

Zhinan Chen

China

Benjamin Chen

USA

Jianquan Chen

USA

Jianzhu Chen

USA

Chao Cheng

USA

Mario Chiariello

Italy

Kazuyoshi Chiba

Japan

Diana Chu

USA

Kwang Chul Chung

South Korea

Simon Chuong

Canada

Audrey Claing

Canada

Maria Lucia Correa-Giannella

Brazil

Ana Cuenda

Spain

Federico Dajas-Bailador

UK

Christian Dani

France

Duane Davis

USA

Ivan Dikic

Germany

Goberdhan Dimri

USA

Adeline Divoux

USA

Dan Dumont

Canada

Stuart Egginton

UK

Paul Evans

UK

Rita Falcioni

Italy

Bertrand Favre

Switzerland

Minghui Gao

USA

Reid Gilmore

USA

Andrew Gladden

USA

Simon Glerup

Denmark

Margarete Goppelt-Struebe

Germany

Mark Gorrell

Australia

Devrim Gozuacik

Turkey

Darren Griffin

UK

Shawn Patrick Grogan

USA

Jörg Großhans

Germany

Reinhard Gruber

Switzerland

Fatima Gyoeva

Russia

Sang Soo Hah

South Korea

Krishnan Harshan

India

Laura Hernandez

USA

Volker Herzog

Germany

James Hickman

USA

Shinji Hirotsune

Japan

Denise Hocking

USA

Liu Hong

USA

Xiaoyu Hu

USA

Yujie Huang

USA

Gareth Inman

UK

Alicia Jawerbaum

Argentina

Janos Kanczler

UK

Nicole Kane

UK

Rakez Kayed

USA

Thomas Kelly

USA

Fadi Khasawneh

USA

Scot Kimball

USA

Tomoshige Kino

USA

Etsuko Kiyokawa

Japan

Linda Klavinskis

UK

Hans Knecht

Canada

Viktor Korolchuk

UK

Gerd Krause

Germany

Gary Kupfer

USA

Ki-Sun Kwon

South Korea

Nathalie Lamarche-Vane

Canada

Muriel Lambert

USA

Brigitte Lankat-Buttgereit

Germany

Lawrence Lash

USA

Jeanne Lawrence

USA

Te Chang Lee

Taiwan

Andre Levchenko

USA

Fiona Lewis

UK

Luyuan Li

China

Wolfgang Link

Portugal

Lijun Liu

USA

Yan Liu

USA

Maria Jesús Lopez -Zabalza

Spain

Wei Luan

Australia

Enrico Lucarelli

Italy

Andrew Macdonald

UK

Muniswamy Madesh

USA

Thery Manuel

France

Jiang Maorong

China

Normand Marceau

Canada

Samuel Marguerat

UK

Robert Markus

Sweden

Manuela Martins-Green

USA

Karl Matter

UK

Lindolfo Meirelles

Brazil

Juanita Merchant

USA

Andreas Merdes

France

David Milstone

USA

Yuko Mimori-Kiyosue

Japan

Kensaku Mizuno

Japan

Samarendra Mohanty

USA

Andrew J Mouland

Canada

Keiko Naruse

Japan

Robert Newman

USA

Davis Ng

Singapore

Christopher Nicchitta

USA

Shin Nishimatsu

Japan

Ruifang Niu

China

Josselin Noirel

UK

Kathleen O'Connor

USA

Hideyo Ohuchi

Japan

Monilola Olayioye

Germany

Pedro Fontes Oliveira

Portugal

Lisa Oliver

France

Michael Olson

UK

Yusuke Ono

Japan

Shoichiro Ono

USA

Ann-Kristin Östlund Farrants

Sweden

Tim Palmer

UK

Stephen Parnell

USA

Yash Patel

USA

Andrew Peden

UK

Ioannis Prassas

Canada

Vegesna Radha

India

Mrinalini Rao

USA

Thipparthi Reddy

USA

Heinz Redl

Austria

Taiyoun Rhim

South Korea

Wiltrud Richter

Germany

Francisco Rivero

UK

Bernard Roelen

Netherlands

Simon Rousseau

Canada

Changgeng Ruan

China

Milena Saqui-Salces

USA

Matthias Schweizer

Switzerland

Xiaobing Shi

USA

Puran Sijwali

India

David Soll

USA

Barbara Stecca

Italy

Paul Steimle

USA

Martin Stoddart

Switzerland

Pavel Strnad

Germany

Jorge Suarez

USA

Alexandra Surcel

USA

Ken Suzuki

UK

Yasuhiko Tabata

Japan

Shin'Ichi Takeda

Japan

Jun Jie Tan

Malaysia

Hou-Quan Tao

China

Gianluca Tell

Italy

Sophie Thenet

France

Alexander Theos

USA

Madoka Uezumi

Japan

George Uhl

USA

György Váró

Hungary

Christina Van Itallie

USA

Mangalakumar Veerasamy

India

Alexey Vereninov

Russia

Nuria Vilaboa

Spain

Jamboor Vishwanatha

USA

Shou-Lin Wang

China

Yuping Wang

USA

Gregory Watson

Australia

Irena Weber

Switzerland

Frances Weis-Garcia

USA

Christian Widmann

Switzerland

Christian Wolfrum

Switzerland

Woodring Wright

USA

Xuechun Xia

China

Wenhua Xiao

China

Lixin Xie

China

Lianping Xing

USA

Sidong Xiong

China

Soichiro Yamada

USA

Wei Yuan Yang

Taiwan

Xiaoyong Yang

USA

Bing Yao

USA

Shui Ye

USA

Kyoko Yokomori

USA

Puangrat Yongvanit

Thailand

Ronen Zaidel-Bar

Singapore

Jianhua Zhang

USA

Quansheng Zhou

China

Dov Zipori

Israel

